# A forecasting model for drug utilization and expenditure integrating a Cellular Automata model with the Budget Impact Analysis approach. Preliminary results

**DOI:** 10.1186/2052-3211-8-S1-P9

**Published:** 2015-10-05

**Authors:** Roberta Joppi, Elisa Cinconze, Luca Demattè, Renato Guseo, Claudio Jommi, Cinzia Mortarino, Daniela Pase, Chiara Poggiani, Alessandro Roggeri, Daniela Roggeri

**Affiliations:** 1 Pharmaceutical Department, Azienda ULSS 20, Verona, 37122, Italy; 2 Consorzio Interuniversitario CINECA, Casalecchio di Reno (BO), 40033, Italy; 3 Department of Statistical Sciences, University of Padova, Padova, 35121, Italy; 4 Department of Pharmaceutical Sciences, University of Piemonte Orientale, Novara, 28100, Italy

## Background

The considerable pressure on healthcare systems, exerted by increasing expenditures for new drugs, urges specific initiatives, including the development of new models, to optimize the managed entry of new medicines and guarantee their sustainability.

## Objectives

To develop a forecasting model for drug utilization and expenditure of emerging medicines identified, prioritized and critically assessed by the Italian Horizon Scanning Project (IHSP), integrating a cellular automata (CA) model describing the diffusion process on the market with the budget impact analysis (BIA), performed before the market entry of a new drug.

## Methods

Selection and critical evaluation of high-impact emerging medicines. Development of CA and BIA models for emerging drugs, using medical prescription data from the administrative ARNO-CINECA databases.

## Results

The first-in-class emerging anti-diabetic dapagliflozin was selected and critically evaluated by the IHSP about 12 months before the European Marketing Authorization (MA). Other competitors already on the market were identified. A CA model describing the diffusion process of more than 200 Italian specialties of oral antidiabetic drugs (ATC A10B), sold between 2000 and 2014 has been developed and validated.

A protocol for the identification of the real-world target population in the ARNO-CINECA database was set up on the grounds of the expected indication for dapaglifozin. The estimation of the budget impact of dapagliflozin is ongoing based on the estimation of market shares, through the application of the CA model, the analysis of the identified target population and the analysis of the potential variations in related healthcare costs for the treatment of type 2 diabetes, after the introduction of dapagliflozin.

## Conclusions

The proposed forecasting model (C-ToBIA model) predicts the impact of emerging drugs on the National Health System (NHS), under the sufficient conditions for estimability. The originality of the C-ToBIA model is basically related to the assessment of emerging drugs 12 months before the MA date, and the estimation of the diffusion process and the potential financial impact before market entry.

The C-ToBIA model will help to timely estimate the possible utilization pattern of new medicines and their potential impact on the NHS before their market entry.

